# Photo-sensitivity of large area physical vapor deposited mono and bilayer MoS_2_

**DOI:** 10.1186/s40580-014-0022-6

**Published:** 2014-08-09

**Authors:** Baoming Wang, Christopher Muratore, Andrey A Voevodin, Md Amanul Haque

**Affiliations:** 1 Mechanical & Nuclear Engineering, the Pennsylvania State University, University Park, PA 16802 USA; 2 Chemical & Materials Engineering, University of Dayton, Dayton, OH 45469 USA; 3 Materials and Manufacturing Directorate, Air Force Research Laboratory, Wright Patterson AFB, OH 45433 USA

## Abstract

We present photosensitivity in large area physical vapour deposited mono and bi-layer MoS_2_ films. Photo-voltaic effect was observed in single layer MoS_2_ without any apparent rectifying junctions, making device fabrication straightforward. For bi-layers, no such effect was present, suggesting strong size effect in light-matter interaction. The photo-voltaic effect was observed to highly direction dependent in the film plane, which suggests that the oblique deposition configuration plays a key role in developing the rectifying potential gradient. To the best of our knowledge, this is the first report of any large area and transfer free MoS_2_ photo device with performance comparable to their exfoliated counterparts.

## Correspondence/Findings

Atomically thin semiconductor materials with large band gaps are touted for aggressively scaled electronic and optoelectronic applications [1]. Even though graphene does not have a band gap, its advent has galvanized the research on two-dimensional (2D) materials, notably the transition metal di-chalcogenides (TMD) [2]. The best example is molybdenum di-sulfide (MoS_2_), which has received significant attention in the scientific community due to high carrier mobility, large current on/off ratio and excellent interface quality with the gate dielectrics [3]. Bulk MoS_2_ has an indirect band gap of 1.2 eV which changes to a direct band gap of 1.83 eV for a monolayer MoS_2_ [4,5]. This leads to unprecedented light-matter interaction (high absorption coefficient and efficient electron–hole pair generation under photo-excitation) that has been studied in the form of photoluminescence, photoreponsivity, photoconductivity and photo-voltaics [1,6-10]. While the existing literature on mono or few-layer MoS_2_ opto-electronics promise revolutionary capabilities of next generation devices, all these studies are performed on exfoliated flakes. Since exfoliation is not a sustainable path beyond basic science, there is a critical need for large area growth of mono-layer MoS_2_ and its opto-electronic characterization. This provides the motivation for the present study, where we deposit large area (> cm^2^) mono and bi-layer MoS_2_ through magnetron sputtering and then study the effect of layer number on the interaction of matter and light. Since the deposition is directly performed on the desired substrate, our fabrication processes are performed directly on the specimen. Specimen transfer to another sample is not needed and contamination is avoided. To the best of our knowledge, this is the first report of any large area, transfer free monolayer MoS_2_ photo-voltaic or photo-detector device with performance comparable to their exfoliated counterparts.

**Figure 1:**
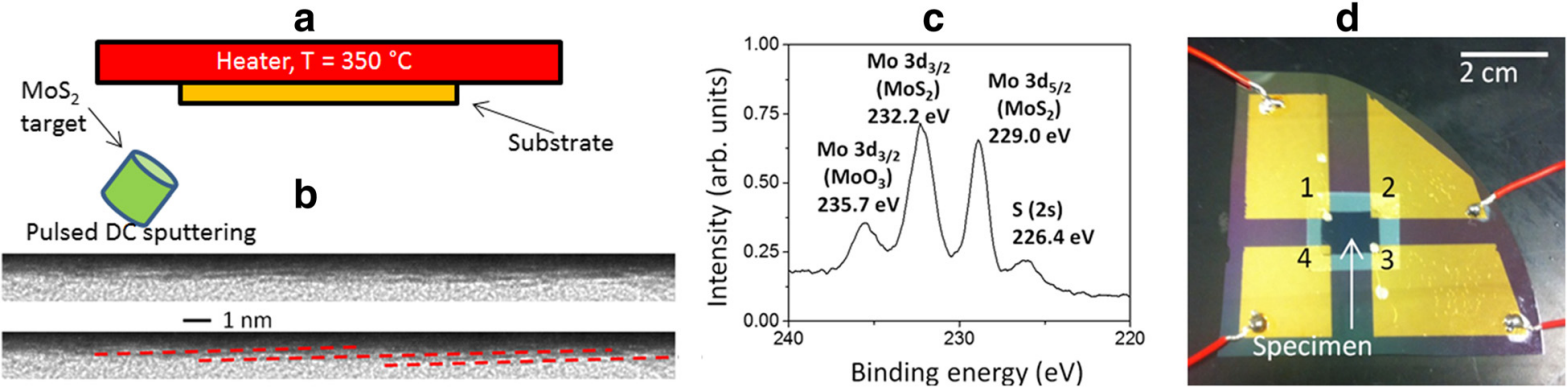
**Experimental settings and characterization of 2D MoS**_**2**_ **films directly grown by sputtering.** **(a)** schematic of the pulsed dc magnetron sputtering configuration **(b)** cross-sectional TEM (dashed lines are added for highlighting the MoS_2_ layers, **(c)** x-ray photoelectron spectroscopy data revealing the structure **(d)** 1 cm × 1 cm MoS_2_ specimen packaged for van der Pauw measurements showing the large active area.

We employed physical vapour deposition with base pressure below 5×10^−9^ Torr for atomically sharp and clean interfaces. The mono and bi-layer specimens were grown on 100 nm thick thermal oxide coated silicon wafer pieces via magnetron sputtering using a solid 3.3 cm diameter MoS_2_ target of 99.95% purity. The substrates were ultra-sonically cleaned prior to introduction via a vacuum load-lock and mounted on an electrically grounded fixture with heating and rotation features. The substrates were then heated to 350°C while ultra-high purity argon gas was introduced at a constant flow rate of 25 sccm to a pressure of 15 mTorr. Figure 1a shows the deposition setup schematically. Figure 1b shows the cross-sectional transmission electron microscopy of the specimens using a FEI Titan microscope for thickness measurements and calibration. The red dashed lines are superimposed on the figure to reveal the parallel atomic layers with likely turbo-stratic structure, where all basal planes are parallel and coplanar, but rotated about the crystals’ z-axes. Or in other words, the specimens are not single crystal layers, but rather a large ensemble of small MoS_2_ grains with uniform thickness and excellent lateral coherence. Specific processing conditions in terms of power modulation to the sputtering cathode were selected based on prior work [11,12] to obtain atomic scale thickness control and very high crystallinity. Both Raman and x-ray photoelectron spectroscopy were then employed to characterize the specimens for thickness and stoichiometry. Figure 1c shows high resolution Mo 3d spectra with Mo peak shift to 229.0 eV indicating the +4 oxidation state for Mo, the hallmark of covalent bonding with sulfur in MoS_2_ [13]. Figure 1c also shows a peak identified at 232.2 eV, which is the 3d_3/2_ peak for Mo and its shift from 231.4 eV also indicates bonding to S.

To verify the continuity and integrity of the deposited films, we prepared specimens with 1 cm × 1 cm active area, whereas the conventional practice is to pattern the semiconducting material as micro to nanoscale strips with metallic contacts as source and drain for a transistor configuration. A physical shadow mask was used to protect the specimen while exposing the four corners, on which 5 nm thick titanium and 100 nm thick gold were evaporated to make the electrical contacts. The chip is then mounted on another wafer piece with large gold plated electrodes using a thermal tape. The four corners of the specimen are then wire-bonded to the larger pads. Figure 1c shows a specimen prepared for van der Pauw characterization [14] of the electrical resistivity under both dark and illuminated conditions. The van der Pauw setup was calibrated with a calibration specimen to achieve ± 5% error limit. Table 1 shows the dark dc conductivity results for the specimens. Such high values of resistivity are due to the fact that ultra-high vacuum condition results in cleaner MoS_2_ surfaces and domain structures. More importantly, the device fabrication process did not require the film to be transferred to a different substrate, so the associated contamination is avoided.

**Table 1:** **Dark dc resistivity of the mono and bi-layer MoS**_**2**_ **specimens**

**Specimen type**	**Specimen 1**	**Specimen 2**
Mono-layer	1.87 × 10^−5^ ohm-cm	1.88 × 10^−5^ ohm-cm
Bi-layer	2.98 × 10^−5^ ohm-cm	2.93 × 10^−5^ ohm-cm

**Figure 2:**
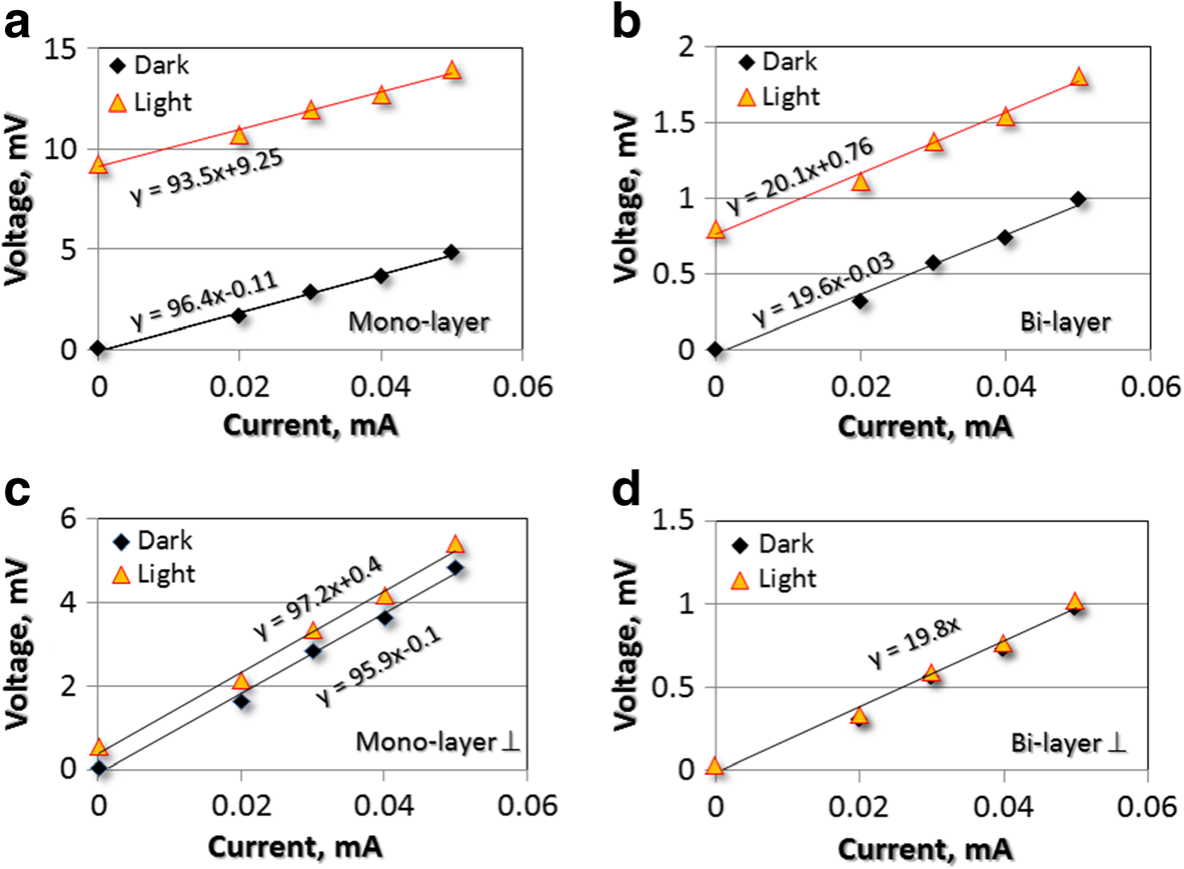
**I-V characteristics under dark and 1.2 milli-Watts illumination for (a) mono-layer and (b) bi-layer MoS**_**2**_ **devices.** The corresponding values, when measured with electrodes at perpendicular (⊥) direction in **(** **c** **)** monolayer and **(** **d** **)** bilayer devices exhibit remarkable anisotropy in photo-sensitivity. Predominant nature of ohmic contact is seen for all the devices.

After dark dc characterization, we illuminated the devices with a 30 W light-bulb located at about 10 cm distance. The measured luminous flux per unit area was measured to be about 24 kLux using a light-meter. Figure 2a and b show the experimental results for the mono and bi-layer specimens. For the mono-layer, we observed the highest open circuit photo induced voltage of about 9.25 mV (without any applied bias). This value is significantly reduced to about 0.75 mV for the bi-layer specimen. These experiments were performed for negative bias to exhibit consistent behaviour. For even thicker specimens, we did not observe any appreciable photo-voltaic effects. We also observe that the effect is extremely direction dependent. For example, Figure 2c and d shows the photo-voltage measurement on the terminals perpendicular to that shown in Figure 2a and b. Such change in direction resulted in dramatic changes in the voltage obtained from the monolayer specimens (Figure 2c) and completely eliminated any such voltage in the bilayer specimens (Figure 2d). Note that in Figure 2d, the dark and illuminated behaviour is almost the same.

A remarkable feature of this study is the apparent ohmic nature of the electrical contacts, as shown in Figure 2. Almost all existing photo-detector devices in the literature [6-9,15,16] present Schottky type contact and the choice of electrode work function is very critical in 2D devices to obtain ohmic contacts. Desired features of the contact are (i) small lattice mismatch at the interface (ii) maximized overlap between the density of states (DOS) at both sides of the interface, (iii) large DOS at the Fermi level throughout the interface region to form delocalized states with low effective electron mass in order to efficiently transfer electrons between the metal and the semiconductor, (iv) a minimized potential barrier at the interface to maximize current injection [17]. Our choice of metals for the electrical contacts therefore appears to be satisfying these conditions. While it is expected that the direct band gap will enhance the photon absorption, formation of an electrical bias through predominantly ohmic contact as seen in Figure 2 is unprecedented. One way to view the observed phenomenon could be the negative-photoconductivity (increase in resistivity under photo-excitation) effect. Essentially, the resistivity of the mono and bilayer specimens increased to 3.5 and 1.1 times respectively under illumination. This is contrary to the conventional observation where light induced charge generation actually increases conductivity. This clearly indicates that the generated charge has been isolated and trapped at the electrodes. To achieve negative photo-conductivity, either a hetero-structure to accept and trap the charge [18-21] or surface plasmon resonance [22] is needed. Both these mechanisms may be partially responsible. For example, if the MoS_2_ specimen is terminated with a Mo layer, it can transport the generated charge to trap it in the oxide substrate of the 2D layers. The obtained experimental results therefore suggest a mechanism for maintaining a potential gradient or asymmetry. While a photo-thermo-electric mechanism has been proposed [1] to be an alternative for conventional Schottky barriers, this is not the case for our study. To prove this, we have varied the location of the light source (along the plane perpendicular to the distance between the source and specimen) and observed that the maximum photo-voltage occurs when the light source is about 45° angles inclined to the vertical. Since this is not the smallest distance from the specimen, we also do not expect this to provide highest photo-thermal energy.

To explain the anomalous observation of photo-voltaic effect, we closely examine the giant photovoltaic (voltages many times larger than the potential drop across the energy gap) effects in thin semiconducting films. The early literature [23] indicates occurrence of this intriguing phenomenon in many materials, however the inconsistency in experimental results has contributed to the lack of understanding and technological relevance. Nevertheless, a consistent observation is that an electro-motive potential gradient (the end closer to the source shows negative polarity) is seen in semiconducting thin films when the insulating substrate is inclined with respect to the incident deposition flux. This is exactly same as our deposition configuration as shown in Figure 1a. Interestingly, this specific condition is impossible to achieve in the conventional techniques (exfoliation, chemical vapour deposition). It is hypothesized that the oblique deposition creates nanoscale p-n junctions due to stacking fault, domain boundaries (Figure 1b suggests turbostratic structure of the films) or the Dember effect [23].

A counter-argument could be that even though the I-V profile is linear and no rectifying junction was intentionally fabricated in the devices, there could be Schottky barriers due to the work function of the Ti electrode [24] or through the localized doping of the MoS_2_ (similar to graphene induced doping [25]). If this were the case, the effect would be the same for all of the four electrodes and there would not be any anisotropic behaviour observed. At the same time, if the fundamental mechanism indeed involves the oblique deposition of the MoS_2_, we would observe large potential between the ends closest and farthest to the deposition source and minimal potential in the perpendicular direction. To corroborate this hypothesis, we measured the open circuit voltage due to illumination by simply changing the electrical terminals in our van der Pauw structure. It is expected that the end of the specimen closest to the deposition source will have negative while the farthest end will have positive polarity. The two other ends that are equidistant to the deposition source should have the same potential and thus will not show any photo-voltaic effect. Or in other words, the observed photovoltaic effects should be highly anisotropic. As shown in Figure 2c and d, this is indeed the case. This evidence corroborates our hypothesis since the MoS_2_ layers are not expected to show in-plane electrical anisotropy of such large magnitude. The literature contains evidence of thermal anisotropy in 2D MoS_2_ [26], where the degree of anisotropy is around 0.8.

We further characterized the monolayer based devices by controlling the illumination through an aperture and exposing only the electrical contact areas. This is commonly observed in 2D opto-electronic devices [1,7,9]. Unfortunately, we were not able to accurately measure the effective area for illumination. According to the literature, the photo-current decays about 2 microns away from the electrical contact. If we conservatively estimate this decay length to be 5 microns, the effective illumination area for the approximately 10 mm long edges for the four electrical contacts is about 5 × 10^−8^ m^2^. For the illumination intensity of 24 kLux, the effective illumination power is estimated to be about 1.2 × 10^−3^ Watts. The highest measured steady state photo-current for the monolayer specimens is about 96.4 μA, which results in photo-responsivity of 0.08 A/W. The extrinsic quantum efficiency (η, ratio of the number of charge carriers generated to the number of incident photons) was calculated to be 0.17 using Equation 1.

1 $$ \upeta = \left( h\mathrm{c}/ e\right)\left(\mathrm{I}/\mathrm{P}\uplambda \right) $$

where *h* is the Planck constant, c the speed of light in vacuum, and *e* the electron charge. The photocurrent, incident power per unit area P, and excitation wavelength are denoted by I, P and λ respectively. In an absolute sense, both photo-responsivity and quantum efficiency values are smaller compared to the MoS_2_ literature. However, these quantities are typically reported for exfoliated flakes and also at micro to nano Watt levels. They nonlinearly decrease with increasing power due to screening of the built-in electric field by the excited electrons in the conduction band [7] or saturation of the trap states at the MoS_2_ or MoS_2_-substrate interface. For example, the highest MoS_2_ photo-responsivity is about 880 A/W at 150 pico-Watts, which monotonically decreases to 2 A/W at 1 micro-Watts [9]. Similarly, quantum efficiency of about 0.2 is reported for exfoliated flakes at 20 micro-Watt power [7]. Given our conservative estimate of the illumination area and power level at milli-Watts range, extrapolation of these performance metrics to the comparable power levels in the literature is extremely encouraging for further research. Currently, we are exploring the role of temperature, carrier concentration and back-gate voltage dependence for the present study.

In conclusion, we present evidence of photo-voltaic effects in mono and bi-layer ultra-high vacuum magnetron sputtered large area MoS_2_ specimens in absence of intentionally fabricated p-n junction or Schottky type contact. The photo-responsivity and extrinsic quantum efficiency were estimated to be 0.08 A/W and 0.17 respectively. We also present evidence of potential gradient in oblique deposition of ultrathin semiconducting films and propose that this gradient is instrumental in generating the photo-voltaic effect in ohmic contacts. While most of the MoS_2_ opto-electronics literature is based on photo-transistors fabricated on exfoliated flakes, the extremely simple design (no need for rectifying junctions) involving large area physical vapour deposited 2D MoS_2_ presented in this study can significantly impact technology development in this area.
